# Real-Time Measurement of Solute Transport Within the Lacunar-Canalicular System of Mechanically Loaded Bone: Direct Evidence for Load-Induced Fluid Flow

**DOI:** 10.1002/jbmr.211

**Published:** 2010-08-16

**Authors:** Christopher Price, Xiaozhou Zhou, Wen Li, Liyun Wang

**Affiliations:** 1 Department of Mechanical Engineering, University of Delaware Newark, DE, USA; 2 Graduate Program in Biomechanics and Movement Sciences, University of Delaware Newark, DE, USA

**Keywords:** BONE ADAPTATION, MECHANOTRANSDUCTION, FRAP, OSTEOCYTE, LCS

## Abstract

Since proposed by Piekarski and Munro in 1977, load-induced fluid flow through the bone lacunar-canalicular system (LCS) has been accepted as critical for bone metabolism, mechanotransduction, and adaptation. However, direct unequivocal observation and quantification of load-induced fluid and solute convection through the LCS have been lacking due to technical difficulties. Using a novel experimental approach based on fluorescence recovery after photobleaching (FRAP) and synchronized mechanical loading and imaging, we successfully quantified the diffusive and convective transport of a small fluorescent tracer (sodium fluorescein, 376 Da) in the bone LCS of adult male C57BL/6J mice. We demonstrated that cyclic end-compression of the mouse tibia with a moderate loading magnitude (–3 N peak load or 400 µɛ surface strain at 0.5 Hz) and a 4-second rest/imaging window inserted between adjacent load cycles significantly enhanced (+31%) the transport of sodium fluorescein through the LCS compared with diffusion alone. Using an anatomically based three-compartment transport model, the peak canalicular fluid velocity in the loaded bone was predicted (60 µm/s), and the resulting peak shear stress at the osteocyte process membrane was estimated (∼5 Pa). This study convincingly demonstrated the presence of load-induced convection in mechanically loaded bone. The combined experimental and mathematical approach presented herein represents an important advance in quantifying the microfluidic environment experienced by osteocytes in situ and provides a foundation for further studying the mechanisms by which mechanical stimulation modulates osteocytic cellular responses, which will inform basic bone biology, clinical understanding of osteoporosis and bone loss, and the rational engineering of their treatments. © 2011 American Society for Bone and Mineral Research.

## Introduction

Osteocytes, the most numerous cells in bone, form a cellular network embedded within the mineralized matrix and are ideally situated to regulate the homeostasis and mechanical adaptation of bone.(1,2) The viability and function of osteocytes require an adequate supply of nutrients (eg, glucose), disposal of waste products (eg, lactic acid), and exchange of endocrine, paracrine, and autocrine regulatory signals (eg, sex hormones, nitric oxide, prostaglandins, cytokines, and growth factors).(1,2) However, simple diffusion of these molecules may not be sufficient for maintaining cell viability and function(3) because (1) osteocytes are embedded in a largely impermeable matrix,(4) (2) solute transport is restricted to the narrow pericellular annular fluid space surrounding the osteocyte cell body and processes (gap < 1 µm)(5), whereas the cell-to-cell spacing is relatively long (∼30 µm)(6), and (3) some osteocytes are found at great distances from the vascular supply (up to 200 to 300 µm)(7). As a potential solution to this problem, Pierkarski and Munro, in their seminal 1977 paper, proposed that load-induced fluid flow within the bone lacunar-canaliculi system (LCS) serves as the primary transport mechanism operating between the blood supply and osteocytes.(3) In the three decades since its publication, the load-induced fluid flow hypothesis has emerged as a well-accepted mechanism not only of bone metabolism but also of bone mechanotransduction.(8,9)

Few measurements are available to directly support the important conceptual phenomenon of load-induced fluid flow despite a large body of indirect evidence, including analytical modeling(3,10–17) and tracer perfusion studies.(18–20) Modeling studies have helped explore the relationships between loading magnitude and frequency, LCS anatomy, and bone fluid flow.(3,13,14,16,21) Nonetheless, the idealized nature of these models, combined with limited knowledge of the LCS cellular architecture and permeability, suggests that these approaches can provide only general approximations of the actual processes occurring within bone. In vivo tracer perfusion studies have demonstrated the positive correlations between mechanical loading and the penetration of tracers within the bone LCS.(18–20) However, these studies provide only static snapshots of tracer localization, lack the temporal dynamics of fluid and solute transport processes, and often are prone to histologic artifacts that could confound the interpretation of results.(22) Measurements of stress-generated streaming potentials(23–25) have provided more direct evidence of macroscopic fluid movement in loaded bone, but the physical size of the probing electrodes limits these studies to exposed surfaces and carries a low spatial resolution beyond the canalicular level.

To overcome the challenges of quantifying transport dynamics at the bone's cellular level, we recently developed a novel in situ imaging approach based on fluorescence recovery after photobleaching (FRAP).(6) By irreversibly photobleaching exogenously injected tracer molecules within individual osteocyte lacunae and recording their subsequent fluorescence recovery, we were the first to directly quantify the diffusion of various molecules within the intact bone LCS in the absence of applied load.(6,26) The feasibility of using FRAP to measure load-induced convection was investigated further in our multiscaled modeling study,(16) where a poroelastic model of an intact murine tibia subjected to cyclic intermittent end compression was combined with a microscopic three-compartment LCS model to simulate hypothetical FRAP experiments under various loading parameters (such as peak load, loading period, resting period, and tracer size). The study suggested that the FRAP technique could be employed to measure load-induced solute convection in intact murine tibia in situ. The goal of this study was to quantitatively measure the dynamic process of solute convection and fluid flow within the LCS of bones subjected to physiologically relevant mechanical loading using the novel FRAP approach.

## Methods

### Specimen preparation

Adult 12- to 16-week-old C57BL/6J (B6) male mice (*n* = 13; Jackson Laboratory, Bar Harbor, ME, USA) were slowly injected via the tail vein with 5 mg of sodium fluorescein (376 Da; Sigma-Aldrich, St Louis, MO, USA) dissolved in 0.5 mL of phosphate-buffered saline (PBS) under inhaled isoflurane anesthesia. Fluorescein was chosen because it approximates the molecular weight of several metabolic compounds [eg, glucose (180 Da) and lactic acid (90 Da)], regulatory agents [eg, nitric oxide (30 Da), prostaglandin (352 Da), estrodiol (272 Da), testosterone (288 Da), and adenosine-5'-triphosphate (507 Da)], and pharmacologic compounds [eg, vitamin-D (385 Da), bisphosphonates (∼250 Da), and corticosteroids (∼400 Da)], all of which are relevant to osteocyte biology.(2) The tracer was allowed to circulate for 30 minutes prior to euthanization by CO_2_ inhalation.(6,26) The left tibia then was harvested and cleansed of soft/adherent tissues. Tibias from eight animals were stored frozen in PBS supplemented with Ca^2+^ and then thawed prior to testing. The remaining five tibias were tested fresh within 0.5 to 3 hours postmortem. The Institutional Animal Care and Use Committee approved this study.

### Integrated loading and confocal imaging apparatus

**Fig. 1:**
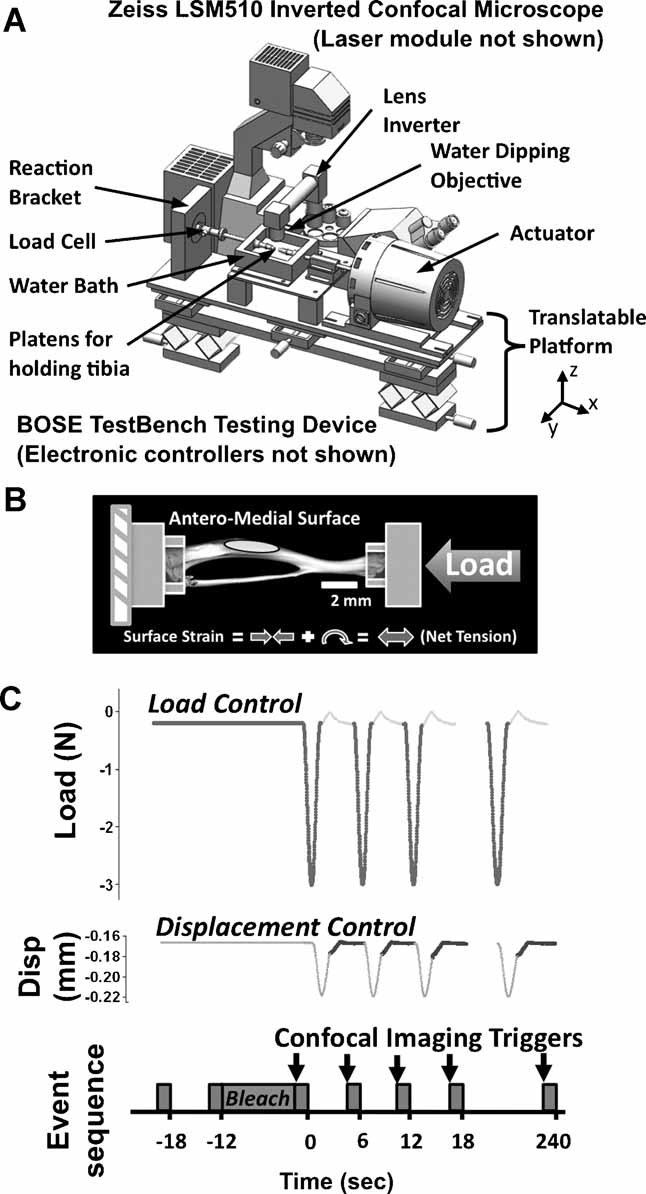
Experimental setup for the mechanical loading of a murine tibia and FRAP imaging of the load-induced solute transport/fluid flow inside the bone LCS. (*A*) The integrated system consisted of an electromagnetic loading device and an inverted confocal microscope. (*B*) Axial end loading of the intact murine tibia and the FRAP imaging region, where a tensile strain was induced due to a combination of bending and compression. (*C*) Synchronization of mechanical loading and FRAP imaging of murine tibiae. *Top row:* A representative trace of the applied load. Thick lines indicate the periods when the loading device was operated in the load control mode. *Middle row:* The corresponding trace of the actuator position. The loading device was switched to the displacement control mode (*thick lines*) during the resting periods when images were acquired between loading cycles. *Bottom row:* The time sequence for executing the three phases (prebleach, photobleaching, and recovery) of the FRAP experiment as well as the imaging trigger signals (indicated by *arrows*) sent from the loading device to the confocal microscope.

In situ mechanical loading and FRAP imaging of murine tibias were performed on a custom apparatus consisting of an electromagnetically actuated loading device (Electroforce LM1 TestBench, Bose Corporation, Eden Prairie, MN, USA) integrated with an inverted confocal laser scanning microscope (Zeiss LSM 510, Carl Zeiss, Inc., Thornwood, NY, USA) (Fig. 1*A*). The loading device, built on a translational platform, was positioned next to the microscope base. A 40×, 0.8 numerical aperture water-dipping lens (W Achroplan, Carl Zeiss, Inc.) was attached to an objective inverter (LSM Technologies, Etters, PA, USA), which was used to direct the imaging path away from the microscope stage onto the specimen immersed in a PBS bath maintained at 37°C (Fig. 1*A*). The tibia was held between customized brass platens (Fig. 1*B*) and positioned such that a relatively flat region of the anteromedial surface of the diaphysis (∼25% to 50% distal from the proximal tibial plateau) was oriented below the objective and parallel to the image plane (Fig. 1*B*). Loads were applied to the distal end of the tibia through the Bose actuator and the proximal tibial plateau was fixed at the reaction bracket (Fig. 1*B*), where the load magnitude was measured using a 5-lb load cell. The position of the actuator head was recorded using a linear variable differential transformer integrated within the actuator. The system had been tested and calibrated using beams machined from engineering and bone materials, and the relationship between applied load and resulting surface strain was quantified in tibias from similarly aged B6 mice previously.(27)

### Loading protocols

Cyclic loads were applied to intact tibiae ex vivo using a rest-inserted end-compression model.(28,29) The tibiae first were subjected to a preconditioning protocol consisting of a tare load (–0.2 N) for 5 minutes, followed by a ramp to –3.0 N at a rate of 0.5 N/s, a 30-second hold at –3.0 N, 10 cycles of compression using a haversine waveform (varying between –0.2 and –3.0 N at 0.5 Hz), and concluding with a 90-second hold at the –0.2 N tare load. This preconditioning protocol was designed (1) to settle the bone into the fixture platens, (2) to reduce tissue creep during subsequent dynamic loading, and (3) to define the actuator position after the preconditioning test, which was the reference position at which the bone was imaged during the cyclic loading tests. Because of the curved shape and viscoelasticity of the mouse tibia, repetitive end compression resulted in discernible creeping of the sample and drifting of the imaging target/window. To overcome this challenge, we alternated the use of load and displacement feedback-control modes to precisely control both the loading magnitude and the position of the actuator and the imaged bone (Fig. 1*C*). Each 6-second loading cycle began with a haversine loading (up to 3.0 N) and unloading waveform with the device operated in the load control mode (*t* = 0 to 2 seconds, Fig. 1*C*). We showed previously that this compressive load induced a peak tensile strain of approximately 400 µɛ on the anteromedial tibial surface due to a combination of bending and compression.(27) The loading device then was switched into displacement control mode during the resting/imaging period (*t* = 2 to 6 seconds), and the actuator was moved to the reference position defined earlier (*t* = 2 to 4 seconds) and held at that position (*t* = 4 to 6 seconds) for image acquisition (detailed below, Fig. 1*C*). This 6-second loading/imaging cycle was repeated 40 times for a total test duration of 240 seconds.

### FRAP imaging protocol

FRAP imaging of intact bone via the confocal microscope has been described previously.(6,26) Briefly, a cluster of fluorescently labeled lacunae approximately 25 to 40 µm below the tibial periosteal surface was identified. A central lacuna then was selected as the target of the FRAP experiment and tightly enclosed with an elliptical region-of-interest (ROI) tool. The imaging setup included a 488-nm laser excitation wavelength, a 505- to 530-nm bandpass emission filter, 512- × 512-pixel images, scanning speed of approximately 1 second/frame, in-plane (*xy*) resolution of 0.22 µm/pixel, and a confocal pinhole of approximately 4.2 to 6.4 Airy unit (optical slice thickness of 8 to 10 µm). The prebleach, photobleaching, and recovery phases of FRAP experiments were performed in synchronization with the intermittent cyclic loading protocol, as detailed below.

### Synchronization of FRAP imaging and mechanical loading

To capture the dynamics of convective transport with high image quality, the FRAP imaging sequence was synchronized with the rest-inserted loading protocol (Fig. 1*C*). First, two prebleach images were acquired (*t* = –18 and –12 seconds) using the MultiTime macro embedded in the Zeiss LSM software under low laser intensity (∼0.4% transmission). The target lacuna (ROI) then was photobleached to approximately 50% of its initial fluorescence intensity using a high laser intensity (100% transmission, 10 to 20 iterations, ∼10 seconds of bleach time). During both the prebleach and photobleaching phases, the samples were held steady under the –0.2 N tare load. Immediately after photobleaching, the MultiTime macro set the confocal microscope to serve as a slave device awaiting trigger signals from the loading device. Meanwhile, the rest-inserted loading protocol was activated manually in the WinTest software of the loading device. A trigger signal initiated the capture of the first recovery image (*t* = –1 to 0 seconds), followed by the first loading cycle (*t* = 0 to 2 seconds) and the 4-second resting period (*t* = 2 to 6 seconds), during which the next postbleach image was acquired. Subsequently, the WinTest software sent an imaging trigger every 6 seconds to synchronize the loading and imaging of the bone until one FRAP trial was completed, ie, the fluorescence of the photobleached lacunae reached a plateau. Both the prebleach and recovery imaging used the same settings (∼0.4% transmission and 1 second/frame).

### Paired loaded and nonloaded FRAP trials

Immediately following each successful rest-inserted loading FRAP trial, one nonloaded FRAP trial was performed on the same lacuna using the same settings, except that only a tare load (–0.2 N) with no dynamic component was applied. This paired experimental scheme was designed to improve the statistical power to detect differences in solute transport between the two loading conditions because it accommodated the intrinsic variability among different anatomic locations and tested samples. At least three different lacunae were tested per bone. Trials that exhibited unusually high autofading (>10%) and those showing vertical drift of the target lacunae were excluded in later analysis.

### Quantification of loaded and nonloaded FRAP trials

A custom FRAP image-analysis program, written in MATLAB (Mathworks, Inc., Natick, MA, USA) and described previously,(6,26) was used to obtain the tracer transport characteristics from the loaded and nonloaded trials. Briefly, sequential 8-bit .tif images from each of the paired FRAP trials were imported into the analysis software. For each trial, the time course of fluorescence intensity [*I*(*t*')] within the photobleached lacuna, including that prior to photobleaching (*I*_0_), immediately after photobleaching (*I*_*b*_), and the postbleach steady-state intensity (*I*_*∞*_), were calculated. Autofading of the tracer fluorescence during imaging of the recovery phase was corrected for in each trial by referencing a lacuna positioned away from the target lacuna and assumed to have a constant intensity. For each trial, the time-dependent intensity data were normalized as


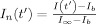
(1)

As shown for FRAP experiments under both diffusion (nonloading)(6) and rest-inserted cyclic loading,(16) the time course of the normalized intensity is predicted to be an exponential function of time (*y = e*^*–t/τ*^) with a characteristic time constant *τ*. The characteristic transport rate *k*, defined as the reciprocal of the time constant (*k* = 1/*τ*), was readily obtained from a linear regression of the experimental intensities with time:


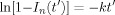
(2)

The characteristic transport rates in the loaded (*k*_load_) and nonloaded/diffusive (*k*_diff_) conditions were determined from the slopes of the initial linear portion of the fitting curves to avoid the accumulation of autofading during the late stage of the recovery.(6,26) The transport enhancement (*k*_load_/*k*_diff_) for each paired test was defined as the ratio of the two transport rates.

### Anatomically based three-compartment LCS transport model

**Fig. 2:**
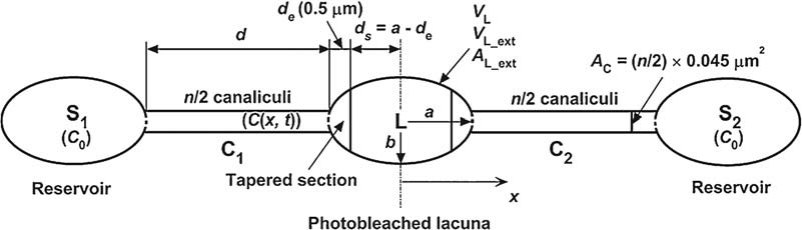
A three-compartment LCS transport model (modified from Zhou et al., 2008). See text for a description of the model parameters; their values are listed in Table 1.

We customized a previously developed three-compartment LCS transport model(16) to estimate the peak canalicular fluid flow velocities in the FRAP experiments. The model consisted of a central photobleached lacuna *L* connected with two neighboring lacunae (modeled as two reservoirs *S*_1_ and *S*_2_) through two sets of *n*/2 canaliculi (*C*_1_ and *C*_2_) (Fig. 2). The photobleached lacuna and canaliculi were modeled as cylinders and smoothly connected with tapered sections (*d*_*e*_). In the model, the cross-sectional area *A* varied along the axial coordinate *x*. Solute concentration *C* was assumed to be dependent on only *x* and time *t*. The model parameters were either measured directly (mean values obtained in the current investigation) or based on empirical values from the literature. Canalicular length *d* and the major and minor radii (*a* and *b*) of the prolate ellipsoidal lacuna were measured directly from the prebleach images in the experiments, from which the lacunar surface area was derived. Contributing canalicular number *n* was calculated based on the lacunar surface area as well as the number density of canaliculi emanating from the lacuna and the fraction of nonphotobleached canaliculi, both of which were quantified in similarly aged mice in our previous study.(6) The lacunar extracellular volume *V*_L_ext_ was calculated based on previous electron microscopic observations that a gap of approximately 1 µm existed between the cell membrane and the lacunar wall,(30) from which an equivalent lacunar fluid cross-sectional area was obtained (*A*_L_ext_ = *V*_L_ext_/2*a*). The pericellular fluid annular area was calculated from that of single canaliculus (0.045 µm^2^) based on previous electron microscopic measures(5) and half the contributing canaliculi number (*A*_*c*_ = 0.045 µm^2^ × *n*/2).

### Calculation of the peak canalicular fluid flow

The paired nonloaded and loaded FRAP experiments can be described mathematically using the diffusion-convection equation:


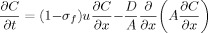
(3)

where *D* is the solute diffusion coefficient, *A* is the cross-section area of the fluid pathway, *u* is the fluid velocity (*u* = 0 for the case of nonloaded FRAP), and *σ*_*f*_ is the reflection coefficient of the tracer in the LCS, which was assumed to be close to 0 for sodium fluorescein.(16) Given values for *D*, *u*, and the initial and boundary conditions, the temporal tracer concentration profiles in the photobleached lacuna were simulated computationally,(16) from which the corresponding transport rates *k* could be calculated using Eq. (2). The peak canalicular fluid velocity in loaded murine tibia was calculated in two steps. First, we simulated pure diffusion (*u* = 0) for a range of *D* values (200 to 300 µm^2^/s) in the model and derived a relationship between *k*_diff_ and *D*. By comparing the experimentally measured *k*_diff_ with the model-simulated results, the solute *D* was determined. Using this *D* as an input, we next simulated the FRAP in the presence of load-induced canalicular flow. The waveform of the flow induced by the intermittent compressive loading was predicted to be approximately sinusoidal.(16) Thus we applied a sinusoidal flow with a peak velocity in the range of *u* = 0 to 120 µm/s and derived the relationship between *u* and the resulting transport enhancement (*k*_load_/*k*_diff_), from which the fluid velocity corresponding to the experimental transport enhancement was obtained.

### Statistical analysis

Both linear and nonlinear fitting of the fluorescence intensity recovery data were performed using the Prism statistical analysis software package (GraphPad Software, La Jolla, CA, USA). Descriptive data are presented as mean ± SD. To determine if rest-inserted loading significantly enhanced the transport of sodium fluorescein within the bone LCS, the mean transport enhancement (*k*_load_*/k*_diff_) was compared with a hypothetical value of 1.0 (indication of zero enhancement) using both a one-sample *t* test and a Wilcoxon signed-rank test. All statistical analyses were performed in GraphPad Prism with a significance level of *p* < .05.

## Results

**Fig. 3:**
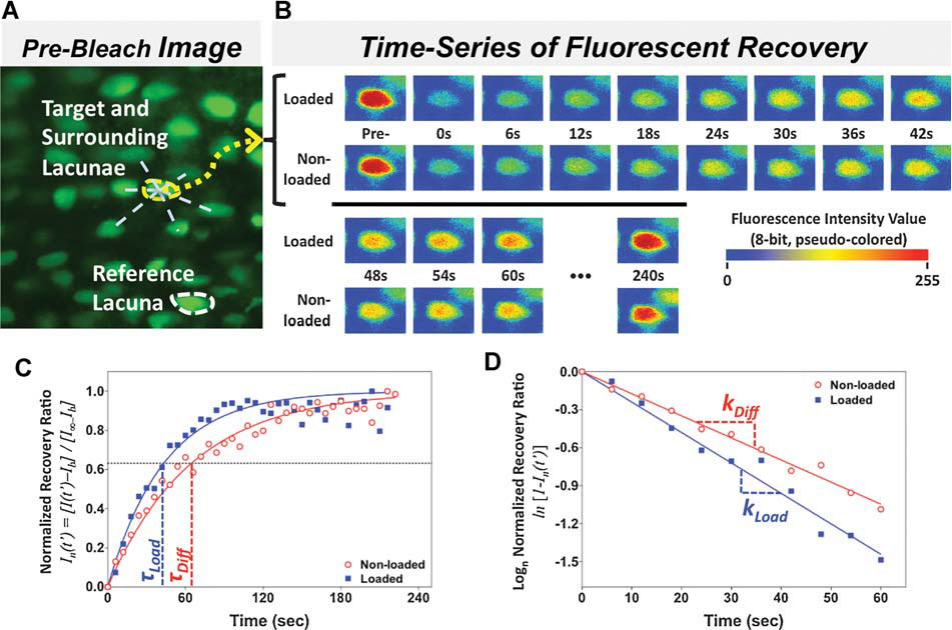
A representative pair of FRAP experiments with sodium fluorescein (376 Da) in a murine tibia subjected to cyclically loaded (peak load of 3 N at 0.5 Hz with a 4-second resting/imaging period between two cycles) or nonloaded paired tests. (*A*) Prebleach image showing a cluster of osteocyte lacunae chosen for FRAP imaging, including the target (outlined in *yellow*) and surrounding lacunae, along with a reference lacuna (outlined in *white*) for autofading correction. (*B*) The time courses of fluorescence recovery within the same photobleached lacuna under loaded or nonloaded conditions. (*C*) Normalized fluorescence intensities *I_n_*(*t*') *=* [*I*(*t*') *– I_b_*]*/*(*I_∞_ – I_b_*) of the paired FRAP trials were fit with a nonlinear regression in the form of *y =* 1 *– e^–t/τ^*. The fluorescence recovery time constant *τ*_diff_ and *τ*_load_ (*dashed vertical lines*) were 65 and 43 seconds (*r*^2^ = 0.97 and 0.93), respectively. (*D*) Transport rates (*k*_diff_ = 0.017/s and *k*_load_ = 0.024/s) were calculated from the slopes of the fitting lines of *y =* ln[*1 – I_n_*(*t*')] versus time (*r*^2^ = 0.99, *p* < .0001 for both). A steeper slope indicated a faster solute transport rate. For this pair of loaded/nonloaded FRAP trials, a transport enhancement *k*_load_/*k*_diff_ of 1.4 was found.

Recovery of sodium fluorescein in the photobleached lacunae was increased by an average of approximately 30% in the presence of intermittent cyclic compression (3 N peak load at 0.5 Hz with a 4-second resting/imaging window) than in the case of diffusion alone. In a representative paired FRAP experiment (Fig. 3), a cluster of lacunae including the photobleached target and reference lacunae were clearly observed in the prebleach image (Fig. 3*A*). High-quality imaging of the region was achieved successfully for both loaded and nonloaded conditions as evidenced by minimal shifting in the image series (Fig. 3*B*). As was typical of all tests, the normalized fluorescence recovery ratio increased exponentially with time in both trials (Fig. 3*C, D*). The shorter recovery time constant [*τ*_load_ (43 seconds) < *τ*_diff_ (65 seconds)] and the higher transport rate [*k*_load_ (0.024/second) *> k*_diff_ (0.017/second)] demonstrated the enhancement of solute transport (*k*_load_*/k*_diff_ = 1.4) due to mechanical loading in this case. No difference in transport enhancement was observed in the loaded/nonloaded tests performed on either previously frozen tibiae (*k*_load_*/k*_diff_ = 1.33 ± 0.23, *n* = 14 pairs) or freshly tested tibial specimens (*k*_load_*/k*_diff_ = 1.30 ± 0.24, *n* = 29 pairs) (unpaired *t* test, *p* = .69; Supplemental Fig. S1). Thus we pooled the data from both groups and found that the applied mechanical loading induced a +31% increase in the transport of sodium fluorescein over diffusion (*k*_load_*/k*_diff_ = 1.31 ± 0.24, *n* = 43 pairs), which was significant when compared with a theoretical *k*_load_*/k*_diff_ value of 1.0 (indication of zero enhancement) using both a one-sample *t* test and a Wilcoxon signed-rank test (*p* < .0001). The reproducibility of the data was reasonable, with intraspecimen coefficients of variation (CVs) ranging from 5% to 31% and interspecimen CVs of 26% and 24% for the nonloaded and loaded trials, respectively. Experimental measures from the freshly tested tibiae are given in Supplemental Table S1.

**Fig. 4:**
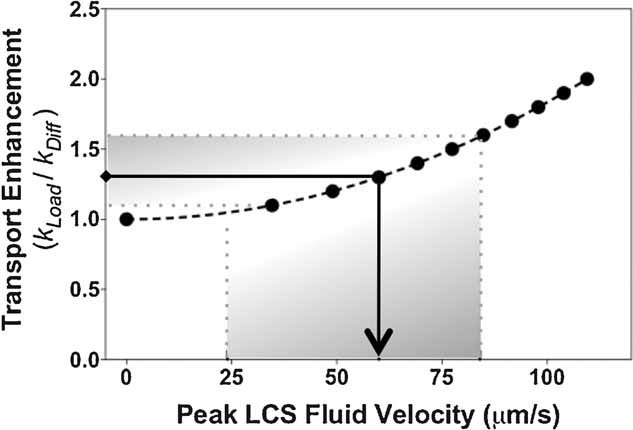
Simulated transport enhancement (*k*_diff_*/k*_load_) as a function of the peak LCS fluid velocity in loaded murine tibia. A power relationship was found between the peak flow velocity and transport enhancement (*k*_load_*/k*_diff_ = 1.0 + 3.7 × 10^−4^ × *u*^1.65^, the *hatched curve*, *r*^2^ = 0.99). A range of flow velocities (24 to 84 µm/s, the *shaded region*) corresponded to the experimentally observed transport enhancements (*k*_load_*/k*_diff_ *=* 1.31 ± 0.24).

The peak canalicular fluid velocity within murine tibiae subjected to rest-inserted compressive loading was found to be on the order of 60 µm/s using the three-compartment LCS model (Fig. 4). The model parameters (listed in Table 1) were chosen based on the mean values of experimental measures from the 29 FRAP tests performed on freshly sacrificed samples. The model-derived relationship between diffusivity and transport rate and that between fluid velocity and transport enhancement are shown in Supplemental Table S2. A diffusivity of 272 µm^2^/s, comparable with values reported previously,(6,26) was found to fit the mean observed nonloaded transport rate (*k*_diff_ = 0.0161/s). A mean velocity of approximately 60 µm/s, with a range of 24 to 84 µm/s, was found to match the measured transport enhancement ratio in the loaded tests (*k*_load_*/k*_diff_ = 1.31 ± 0.24; Fig. 4).

**Table 1:** Experimentally Derived Parameters Used in the LCS Transport Model

Canalicular length	Contributing Canalicular number	Lacunar major radius	Lacunar minor radius	Extracellular lacunar cross-sectional area	Extracellular canalicular cross-sectional area	Loading period	Resting period	Reflection coefficient	Diffusive (non-loaded) transport rate	Loaded transport rate
*d (µm)*	*n*	*a (µm)*	*b (µm)*	*A*_*L*_ *(µm*^*2*^*)*	*A*_*C*_ *(µm*^*2*^*)*	*T (s)*	*R (s)*	*σ*_*f*_	*k*_*Diff*_ *(s*^*−1*^*)*	*k*_*Load*_ *(s*^*−1*^*)*
30	14	8.7	3.9 µm	16.3	0.315	2	4	0	−0.0161	−0.0203

## Discussion

Over the last 30 years, load-induced fluid flow within the bone LCS has emerged as an important transport enhancement mechanism between the vasculature and osteocytes as well as a potent mechanical stimulation to bone cells. Despite its physiologic importance, direct quantification of bone fluid flow under physiologically relevant loading conditions has been lacking. In this investigation, we integrated confocal imaging with mechanical loading and successfully applied FRAP to measure and compare, in real-time and nondestructively, solute diffusion and convection within the LCS of intact adult murine tibiae. We demonstrated that the application of intermittent compressive loads (3 N peak load at 0.5 Hz inserted with 4-second resting periods) could significantly enhance the transport of a small-molecular-weight tracer (sodium fluorescein, 376 Da) in the bone LCS via load-induced convection. This result demonstrated the presence of fluid flow within the lacunar-canalicular system of loaded bone. Based on the experimental transport data, we predicted a significant flow (∼60 µm/s) occurring in the osteocytic canaliculi under the studied conditions. The framework developed herein represents an important advance in quantifying the in vivo microfluidic environment experienced by osteocytes, a key player in bone mechanotransduction and homeostasis.

The significant (31%) transport enhancement found for sodium fluorescein under cyclic loading with a moderate-magnitude (∼400 µɛ) surface strain confirms the physiologic importance of load-induced fluid flow in bone metabolism. Many nutrients, signaling molecules, and therapeutic agents have similarly small molecular weights, including glucose (180 Da), lactic acid (90 Da), nitric oxide (30 Da), estrodiol (272 Da), testosterone (288 Da), prostaglandin (352 Da), adenosine-5'-triphosphate (507 Da), vitamin D (385 Da), bispohophonates (∼250 Da), and corticosteroids (∼400 Da). Since the in vivo metabolic rates for osteocytes are largely unknown, it is difficult to quantitatively compare cellular demands with the diffusive and convective transport measured herein. However, our data suggest that dynamic loading enhances the perfusion of these molecules in bone tissue, potentially allowing embedded osteocytes, especially those located far from capillaries or in interstitial areas, where diffusion is relatively limited, to more readily access these important molecules. To detect load-induced transport enhancement, we adopted a paired experimental design that allowed us to quantify and compare solute transport kinetics in the same LCS locations under both loaded and nonloaded conditions, thereby greatly minimizing the effects of variability among different samples and locations. Because the loading regime in this investigation (intermittent cyclic loading with inserted resting periods) was quite different from that of previously published studies, including mathematical modeling,(3,10–17) investigations of stress-induced streaming potentials,(25,31) and in vivo tracer perfusion studies,(18–20) the quantitative results from this study cannot be compared directly with those studies. However, a recent study(32) demonstrated a qualitatively similar transport enhancement due to transverse compression of the knee using FRAP.

This study represents a major advance in bone fluid flow measurements, providing an empirically based estimate of load-induced fluid flow within the osteocyte canaliculi. Combining FRAP transport data with computational modeling, we were able to predict a peak canalicular fluid flow velocity on the order of approximately 60 µm/s for the given loading conditions. Because experimentally measured solute transport kinetics were used to validate the model output, this approach provided unique advantages over previous modeling efforts. Specifically, the need to specify the bone's poroelastic material properties, such as the relative compressibility between the solid and fluid phases and Darcy's permeability, was eliminated. In the pioneering work of Piekarski and Munro, transport was modeled at the osteonal level in a construct of concentric porous mineral cylinders separated by a fluid phase, where canalicular details were not included.(3) Kufahl and Saha later developed a model including the lacunar-canalicular porosity, but the cell processes and pericellular matrix were not considered.(10) Weinbaum and colleagues presented a series of increasingly refined models that incorporated many anatomic features, including the cell body, cell process, cytoskeleton, and pericellular matrix, to predict fluid flow, streaming potential, and hydrodynamic drag.(12,31,33–35) Other studies have included a variety of architectures, loading and boundary conditions, and diffusivity and permeability parameters.(11,13,15–17,21) In general, these previous models used highly idealized microstructures and theoretical analysis to derive the key parameter (eg, LCS permeability) for determining the fluid movement under mechanical loading. In contrast, our three-compartment transport model, incorporating experimentally measured anatomic features (Table 1), was constructed based on the simple mass-conservation principle. With the canalicular fluid velocity as the model input, the relationship between canalicular flow and lacunar transport enhancement was derived using the well-established diffusion-convection equation. Two predictions from the three-compartment model(16) were that (1) the tracer concentration in the central lacuna recorded at the end of each loading cycle would increase exponentially as a function of time in both loaded and nonloaded FRAP cases and (2) the time constant would be shorter for the loaded case. Both predictions were confirmed in the current FRAP experiments (Fig. 3), suggesting that the model captured the physics of the fluid flow in loaded bone. Most important, we ran two simulations based on the paired nonloaded and loaded FRAP experiments. The tracer diffusivity found from the nonloaded experiments was comparable with values from previous studies, further validating the use of the three-compartment model. Because the model was built with few assumptions of tissue properties, incorporated anatomic measures of the LCS structure, and was validated with experimental measurements of solute transport, the accuracy of the flow velocity estimate was ensured.

The magnitude of flow velocity can explain qualitatively the transport enhancement recorded in the paired FRAP experiments. Given a peak canalicular fluid flow velocity *u* of 60 µm/s in loaded bone, the movement of the front of a solute with a diffusivity *D* of 272 µm^2^/s during a half loading cycle (ie, the solute stroke displacement) could be as large as *d*_*L*_ = 54.5 µm, a summation of the convective displacement *u/*(*πf*) = 38 µm and the diffusive displacement *d*_*D*_ = √(*Dt*) = 16.5 µm for *f* = 0.5 Hz and *t* = 1 second. In contrast, in the nonloaded case, the distance that the solute front moves during the same time period is *d*_*D*_ only. Since the load-induced solute stroke displacement *d*_*L*_ is approximately 180% of the average canalicular length (30 µm) between adjacent lacunae, the fluid flux should enter the larger lacuna as a jet, resulting in solute mixing and a concentration difference (asymmetry) between the influx and outflux to and from the lacuna.(14) Transport enhancement due to such lacunar mixing has been shown in previous theoretical studies(14,16) and could account for the experimental observations in this study.

The magnitude of the load-induced canalicular flow found in this study has important implications in osteocyte mechanobiology. A previous modeling study(12) predicted that the load-induced flow profile in a canaliculus filled with a pericellular matrix was that of a plug-flow containing a thin nonslip boundary layer with its thickness on the order of the effective pore size. Using the experimentally measured effective pore size of 12 nm,(22) a fluid flow of 60 µm/s is expected to impart a fluid shear stress (FSS) on the cell membrane as high as 5 Pa (ie, 50 dyn/cm^2^). While this FSS, resulting from a surface strain of 400 µɛ, is 2.5-fold higher than previous model predictions (∼2 Pa for 1000 µɛ),(12) recent studies suggest that cells, specifically endothelial cells in large blood vessels, may be subjected to such high FSS in vivo.(36) Although most in vitro cell culture studies on osteocytes have used FSS around 2 Pa,(8) some studies demonstrated that osteocytes can tolerate FSS approaching the magnitudes described here.(37) We are fully aware that, like any model-based prediction, the current velocity estimate is sensitive to the choices of model parameters. Our previous study(21) demonstrated that an increase in the canalicular number resulted in a linear increase in the lacunar transport rate, whereas an increase in the canalicular length accompanied a nonlinear decrease in the lacunar transport rate. In this study, all model parameters were derived experimentally (Table 1), except for the number of contributing canaliculi, which was calculated based on the surface area of the lacuna measured in the experiments and the number density of the canaliculi and shape of photobleaching laser beam measured in a separate study.(6) An underestimation of the number of contributing canaliculi would result in an overestimation of the flow velocity. Given the limited resolution in our confocal system, individual canaliculi remain quite difficult to visualize. We hope that future advances will permit visualization of such fine features and further increase the accuracy of the flow estimates.

There are several limitations in this study. Although use of live mice or fully intact limbs with attached soft tissues would have been preferred, pilot tests showed that extensive soft tissue deformation and drifting of the bone under mechanical loading made it impossible to record FRAP in those preparations. Our use of dissected bones and ex vivo testing of postmortem samples raised two potential concerns: (1) whether the absence of circulation in these samples affected solute transport in the loaded and unloaded conditions and (2) whether significant degradation of cells and/or the pericellular matrix within the LCS occurred due to postmortem changes. These concerns were alleviated through a series of experiments in a parallel investigation.(38) In that study, we demonstrated that the contribution of normal vascular pressurization in live, anesthetized mice to the transport of sodium fluorescein through the bone LCS was minimal compared with diffusion.(38) We also demonstrated that repeated photobleaching, postmortem delays in testing (up to 1 hour),(38) or specimen freezing (this study) did not significantly alter sodium fluorescein transport within the LCS. Therefore, we anticipate similar results in live bone as demonstrated in this ex vivo study. Additional limitations include the investigation of loading-enhanced transport for one relatively small molecule using a single loading protocol (ie, load magnitude, load frequency, and rest period were not varied). Molecules of a wide range of molecular weights play important roles in osteocyte biology and cell signaling.(1,2) In solution, larger molecules with smaller diffusivity are expected to experience greater degrees of transport enhancement due to convection. However, the transport pathway in the bone LCS is filled with pericellular matrix that serves as a molecular sieve hindering mobility, especially for larger molecules. A complex biphasic behavior for the convective transport enhancement has been predicted within bone, depending on molecule-matrix interactions, LCS anatomic variations, and loading parameters.(16,21) Lastly, while we measured load-induced solute transport directly via observable changes in tracer concentration, subsequent calculation of the induced canalicular fluid velocity still required model simulation and data fitting.

Despite these limitations, this investigation provided direct evidence for load-induced fluid flow in intact, physiologically loaded bone specimens and clearly demonstrated the proof-of-concept of a novel in situ approach for future fluid-flow studies. By integrating the FRAP technique with an intermittent, rest-inserted, mechanical loading protocol, we developed a real-time, nondestructive, and minimally invasive method for measuring dynamic transport processes in situ. This investigation demonstrated that application of a moderate-magnitude cyclic load (3 N peak compressive load or ∼400 µɛ peak surface strain at 0.5 Hz) with 4-second inserted rest periods to the intact murine tibia resulted in a 31% enhanced transport of a small-molecular-weight tracer sodium fluorescein through the bone LCS. Numerical simulation of the paired loaded/nonloaded FRAP experiments in an anatomically based three-compartment LCS model revealed that a peak canalicular fluid flow of 60 µm/s accounted for the observed transport enhancement. This study convincingly demonstrated the role that mechanical loading plays in bone metabolism and constitutes a direct confirmation of the presence of load-induced fluid flow in the LCS, a potent stimulus to osteocytes. As osteocytes become increasingly important in delineating the mechanotransduction mechanisms in bone,(1,2) the approach developed herein will be a valuable tool to quantify the microenvironment experienced by osteocyte in vivo. These studies are anticipated to help inform our basic biology and clinical understanding of bone fragility, bone loss, and their treatments.
