# Supplementary material for: Real-Time Measurement of Solute Transport Within the Lacunar-Canalicular System of Mechanically Loaded Bone: Direct Evidence for Load-Induced Fluid Flow
Source: J Bone Miner Res. 2010 Aug 16;26(2):277–85. doi: 10.1002/jbmr.211 (PMC3179346; doi:10.1002/jbmr.211)
Supplement: Supplementary file 1 [file jbmr0026-0277-SD1.doc]

**Supplemental Data**


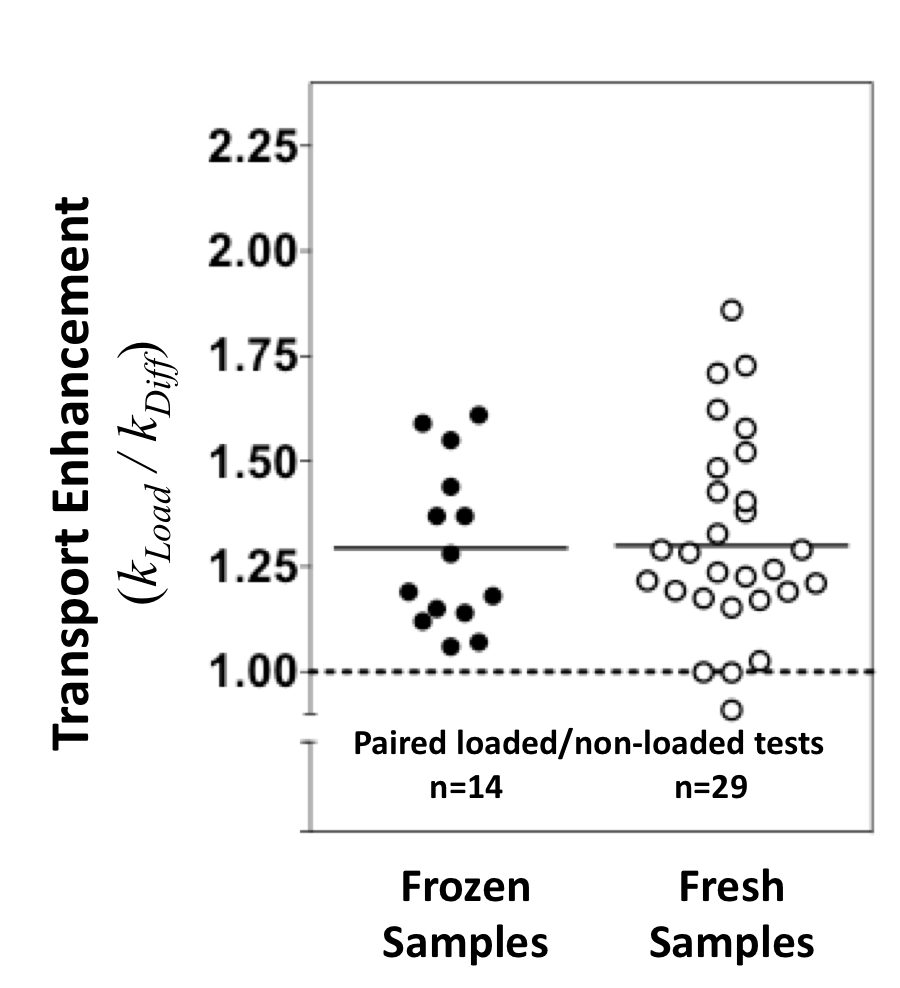


**Fig. 1S:** Transportation enhancements (*k_Load_/k_Diff_*) of sodium fluorescein tested in either previously frozen or freshly sacrificed (0.5−3.0h *post mortem*) intact murine tibiae subjected to the rest-inserted loading. The mean transport enhancement values for the frozen and fresh sample groups (1.33±0.23 and 1.30±0.24, respectively) were not significantly different (p=0.69, unpaired t-test).

**Table 1S: Paired FRAP experiments performed on the freshly tested tibiae**


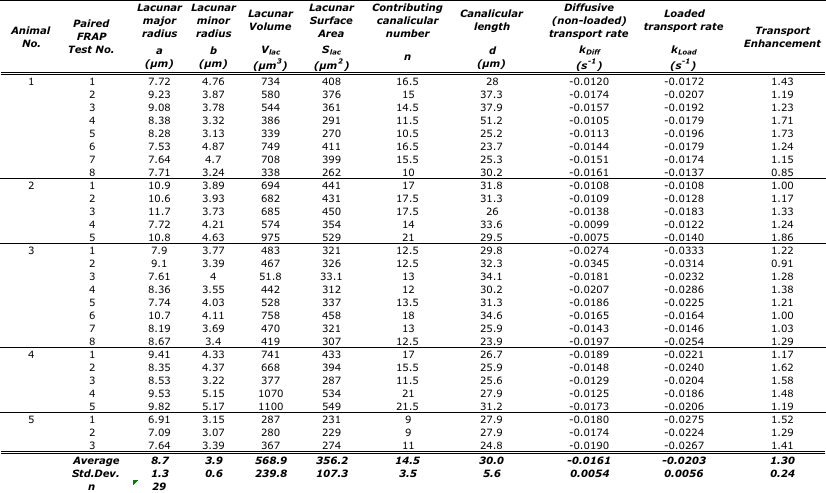


**Table 2S**: Simulation derived relationship between **a)** the diffusion coefficient (*D*) and diffusive transport rate (*k_Diff_*) and **b)** the peak fluid velocity (*u*) and transport enhancement (*k_Load_*/*k_Diff_*).


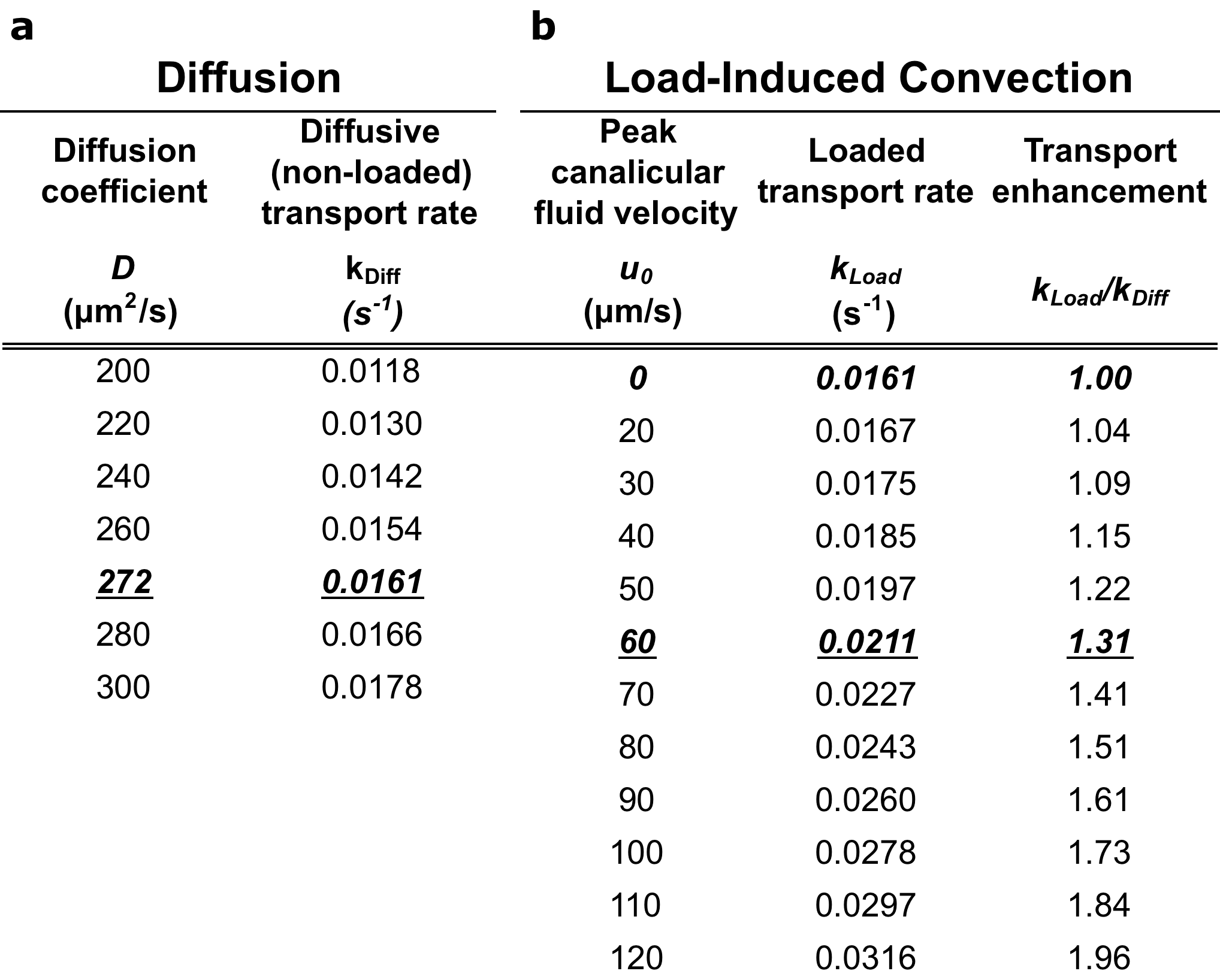


Based on the experimental data *k_Diff_*=0.0161s-1 and *k_Load_*/*k_Diff_*=1.31, the diffusivity and peak canalicular flow velocity were identified (bold and underlined rows).
